# Interaction of the Anticancer Plant Alkaloid Sanguinarine with Bovine Serum Albumin

**DOI:** 10.1371/journal.pone.0018333

**Published:** 2011-04-06

**Authors:** Maidul Hossain, Asma Yasmeen Khan, Gopinatha Suresh Kumar

**Affiliations:** Biophysical Chemistry Laboratory, Indian Institute of Chemical Biology, Council of Scientific and Industrial Research (CSIR), Kolkata, West Bengal, India; University of South Florida College of Medicine, United States of America

## Abstract

**Background:**

Interaction of the iminium and alkanolamine forms of sanguinarine with bovine serum albumin (BSA) was characterized by spectroscopic and calorimetric techniques.

**Methodology/Principal Findings:**

Formation of strong complexes of BSA with both iminium and alkanolamine forms was revealed from fluorescence quenching of sanguinarine. Binding parameters calculated from Stern-Volmer quenching method revealed that the neutral alkanolamine had higher affinity to BSA compared to the charged iminium form. Specific binding distances of 3.37 and 2.38 nm between Trp 212 (donor) and iminium and alkanolamine forms (acceptor), respectively, were obtained from Forster resonance energy transfer studies. Competitive binding using the site markers warfarin and ibuprofen, having definite binding sites, demonstrated that both forms of sanguinarine bind to site I (subdomain IIA) on BSA. Sanguinarine binding alters protein conformation by reducing the α-helical organization and increasing the coiled structure, indicating a small but definitive partial unfolding of the protein. Thermodynamic parameters evaluated from isothermal titration calorimetry suggested that the binding was enthalpy driven for the iminium form but favoured by negative enthalpy and strong favourable entropy contributions for the alkanolamine form, revealing the involvement of different molecular forces in the complexation.

**Conclusions/Significance:**

The results suggest that the neutral alkanolamine form binds to the protein more favourably compared to the charged iminium, in stark contrast to the reported DNA binding preference of sanguinarine.

## Introduction

**Figure 1:**
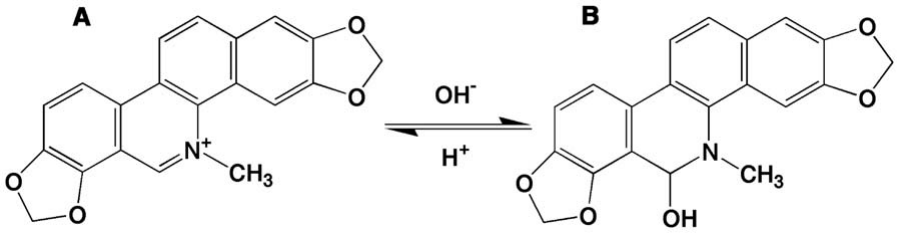
Chemical structure of sanguinarine. (A) iminium form (B) alkanolamine form.

Alkaloids are secondary metabolites of plants especially noteworthy for their remarkable biological activities and potential medicinal use. Plant alkaloids are abundant in nature and have relatively low toxicity to humans compared to other chemicals. Sanguinarine (Figure 1), is a quaternary benzophenanthridine alkaloid, found in many botanical species [1]. It is also a component of argemone oil, responsible for ‘epidemic dropsy’ [2]. Sanguinarine exhibits multiple pharmacological effects, including remarkable antimicrobial and anti-inflammatory properties [3], [4], and is a potential lead compound in cancer therapy inducing apoptosis in a variety of cancer cells through different mechanisms [5]–[11]. Binding of sanguinarine to various DNA and RNA structures and inhibition of the enzyme topoisomerase have also been linked to its pronounced anticancer activity [12]–[16]. Structurally, sanguinarine can exist as the cationic iminium (Figure 1A) and neutral alkanolamine (Figure 1B) forms with a pK_a_ of 7.4 [17]. The nucleic acid binding form has been the charged iminium [18]. Although DNA and RNA binding of sanguinarine has been studied extensively [12]–[20], binding to proteins is yet to be investigated. Effectiveness and efficacy of sanguinarine in therapeutic applications may be modified on the basis of its interaction with serum albumins that may be critical for understanding its toxicity and distribution in cells. Thus, to understand and exploit the pharmaceutical benefit of sanguinarine and to provide the molecular basis of drug action, its interaction with protein must be understood.

Serum albumins, the most multifunctional transport proteins and a major constituent of blood plasma, have many physiological functions and facilitate the transportation and disposition of several endogeneous and exogeneous compounds including proteins and fatty acids to specific targets [21]–[23]. Bovine serum albumin (BSA) is a globular heart-shaped protein, a 583 amino acid residue monomer of 66.4 kDa composed of three structurally similar domains (I, II and III), each containing two subdomains (A and B) stabilized by 17 pairs of disulfide bridges [23], [24]. BSA considerably contributes to colloid osmotic blood pressure and participates in the transport and distribution of many metabolites. Ligand sequestration and transport by BSA impact many functions like metabolism, membrane penetration, half life and other pharmacokinetic properties. BSA has an excellent structural homology of about 80% and repeating pattern of disulphide bonds with human serum albumin. Many studies have revealed that targeted delivery of ligands by BSA is effected through discrete binding sites in the hydrophobic cavities located in subdomains IIA and IIIA in the three structurally similar α-helical domains of the protein [25], [26]. A number of studies on the interaction of small molecules and drugs to BSA have been recently reported [27]–[34] and elucidation of the molecular aspects of the binding continues to be of great importance from the standpoint of understanding protein structure-function on the one hand and designing drug therapy and design on the other.

Here, we present a detailed study on the binding of sanguinarine iminium and alkanolamine forms to bovine serum albumin from a variety of biophysical experiments.

## Results and Discussion

### Absorption spectral study of the interaction

**Figure 2:**
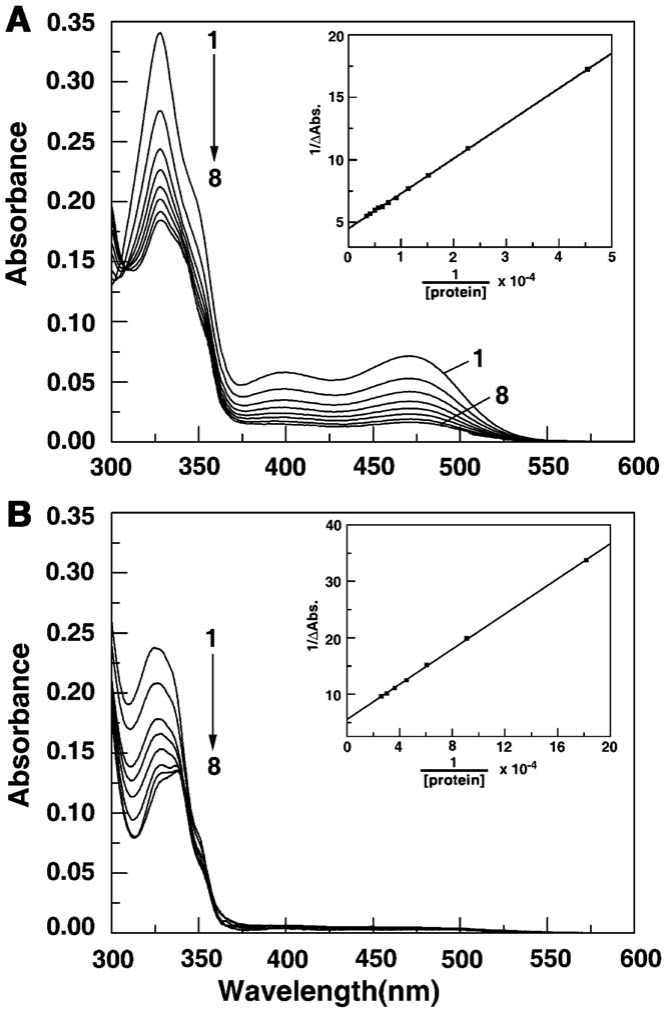
Representative absorption spectra of sanguinarine in presence of BSA. (A) SI (11.06 µM) treated with 0, 22.12, 44.24, 66.36, 88.48, 110.6, 132.72, 154.84 µM (curves 1–8) of BSA and (B) SA (10.94 µM) treated with 0, 5.5, 11, 16.5, 22, 27.5, 33, 38.5 µM (curves 1–8) of BSA. Inset: Respective Benesi-Hildebrand plots for binding.

Sanguinarine iminium and alkanolamine forms have characteristic absorption spectra in the range 300–550 nm that could be monitored to understand the binding to BSA. There are two peaks in this region for SI, a sharp one with maximum around 327 nm and a broad one with maximum around 470 nm. For SA, there is only one peak with maximum around 327 nm (see Figure 2B, curve 1). Results of absorption spectral titration of constant concentrations of SI form (11.06 µM) and SA form (10.94 µM) with increasing concentration of BSA are presented in Figures 2A & 2B. Binding resulted in quenching of the peak intensities with saturation observed at D/Ps (drug/protein molar ratio) of 18 and 3.5, respectively, for SI and SA. The spectral data were analyzed by Benesi-Hildebrand plot (inset of Figure 2A & 2B) to determine the equilibrium constant using the equation,
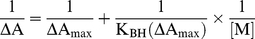
(1)The binding constants evaluated yielded *K*_BH_ values of 1.6×10^4^ M^−1^ for the SI form and 3.46×10^4^ M^−1^ for the SA form. These results suggest a twofold higher affinity of SA to BSA over that of SI.

### Fluorescence spectral study

**Figure 3:**
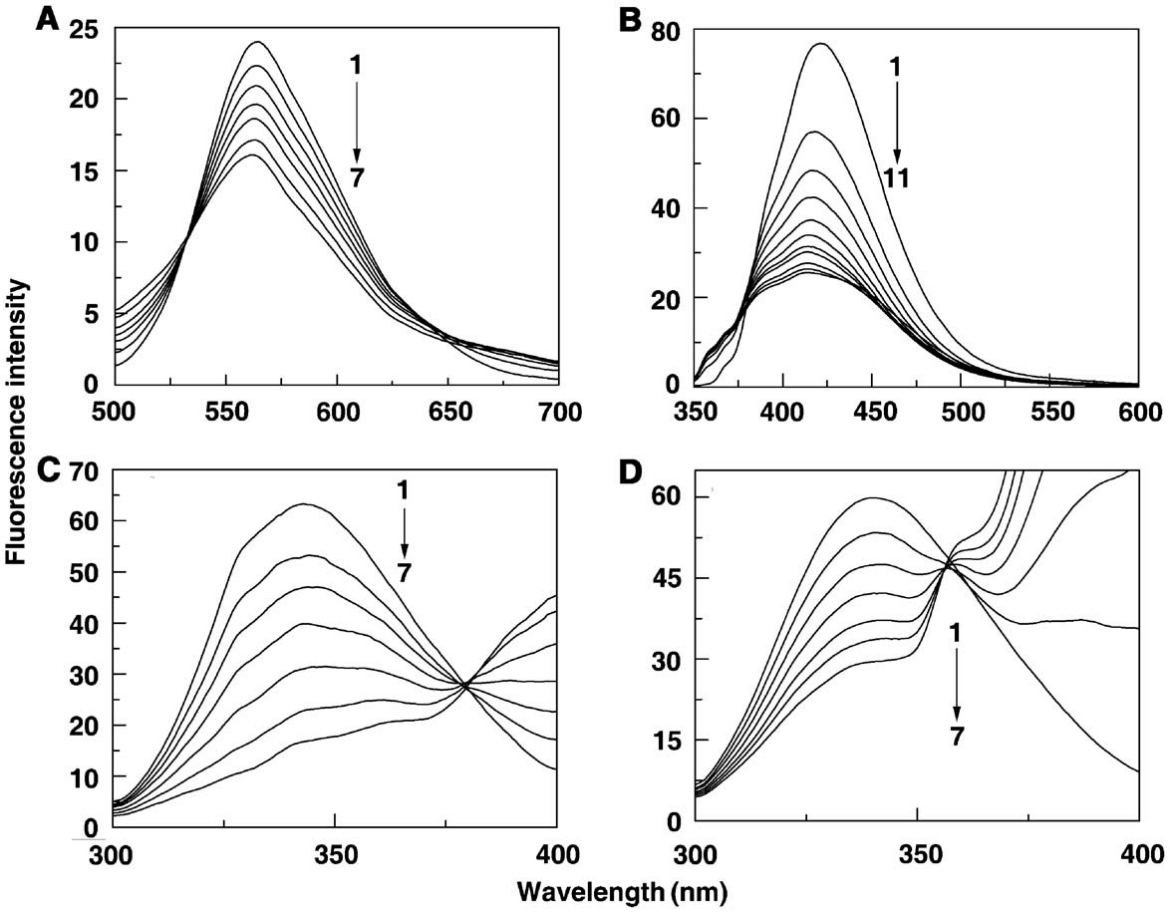
The fluorescence spectra of (a) SI (1 µM) and (b) SA (1 µM) treated with BSA. In panel (A) curves (1–7) denote 0, 1, 2, 4, 6, 8 and 12 µM of BSA, and panel (B) curves (1–11) denote 0, 0.2, 0.4, 0.6, 0.8, 1.0, 1.2, 1.4, 1.6, 2.0 and 2.8 µM of BSA, respectively. Fluorescence spectra of BSA (1 µM) treated with different concentrations of (C) SI and (D) SA. In panel (C) curves (1–7) denote 0, 4, 6, 10, 16, 24, and 32 µM of SI and panel (D) curves (1–7) denote 0, 0.3, 0.6, 0.9, 1.2, 1.5, 1.8 µM of SA, respectively.

To understand the effect of sanguinarine complexation on the protein, the fluorescence of the tryptophan moiety of the protein was monitored by exciting at 295 nm. BSA contains two tryptophan (Trp) residues that possess intrinsic fluorescence [35], [36]. Trp 134 in the first domain is located on the surface in the hydrophilic region of the protein while Trp 212 in the second domain is located within a hydrophobic binding pocket [35]. It has been reported that Trp residues inside the proteins are characterized by a shorter wavelength emission maximum around 340 nm while those on or near the surface of a protein are characterized a longer wavelength emission maximum [37]. Thus, the fluorescence emission of BSA with maximum around 345 nm when excited at 295 nm comes from the Trp residue at 212. Binding of both SI and SA forms leads to decrease in the fluorescence intensity with slight red shift, which may be due to a variety of interactions like excited state reactions, molecular rearrangements, energy transfer, ground state complexation and collision quenching [38]. The fluorescence quenching data is presented in Figures 3A & 3B. At wavelengths beyond 350 nm, both SI and SA forms have strong emission maxima (*vide infra*) and hence these regions are not presented. Saturation was achieved in both cases. The occurrence of isoactinic points at 379 nm for SI and 357 nm for SA form indicated the existence of free and bound forms of sanguinarine in equilibrium. The quenching data was quantified by Stern-Volmer equation
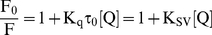
(2)where F_o_ and F are the fluorescence intensities in the absence and in the presence of the quencher, respectively. *K*_q_ is the quenching rate constant. *K*_sv_ is the dynamic quenching rate constant, τ_o_ is the average lifetime of the protein without the quencher, which is of the order of 10^−8^ s [39], and [Q] is the concentration of the quencher. Dynamic quenching refers to a process where the fluorophore and the quencher come into contact during the lifetime of the excited state. Static quenching, on the other hand, refers to fluorophore-quencher complex formation inthe ground state. The plot of F_o_/F versus [Q] was linear (not shown), which is indicative of one type of quenching. The values of *K*_sv_ derived from these plots were 7.58×10^4^ L. mol^−1^ and 3.65×10^5^ L. mol^−1^ for the binding of SI and SA forms at 25°C (Table 1). *K*_q_ calculated from the ratio of *K*_sv_ and τ_o_ showed value of 7.58×10^12^ and 3.65×10^13^ L mol^−1^ s^−1^, respectively, for SI and SA. For dynamic quenching, the maximum value of the scattering collision quenching constant *K*_diff_ is 2.0×10^10^ L mol^−1^ s^−1^ [40]. Since the value of the biomolecular quenching is higher, it can be assumed that the complex formation between the quencher and the protein is by a static mechanism. The static quenching mechanism was further confirmed from the temperature dependence of the quenching; *K_sv_* values decrease with increasing temperature for static quenching while the reverse is observed for dynamic quenching [38]. The fluorescence data were analyzed at three different temperatures, viz. 15, 25 and 35°C. The *K*_sv_ values at the three temperatures are presented in Table 1. The Stern-Volmer quenching constant *K*_sv_ and the quenching rate constant *K*_q_ values (not shown) decreased with increasing temperature, suggesting that the quenching mechanism was due to complex formation rather than dynamic collision. In other words, quenching of the fluorescence of BSA by both SI and SA forms is due to specific complex formation; dynamic collision effects, if any, shall be negligible.

**Table 1:** Binding constants of sanguinarine iminium and alkanolamine forms to BSA derived from the Stern-Volmer method at different temperature.

Sanguinarine	Temperature (K)	*K_sv_* / 10^−4^ L. mol^−1^
Iminium	288	8.74
	298	7.58
	308	6.17
Alkanolamine	288	51.7
	298	36.5
	308	25.0

All the data presented are average of four determinations. The experiments with iminium were performed in citrate-phosphate buffer of pH 6.4 and that with alkanolamine form were performed in carbonate-bicarbonate buffer of pH 9.2.

The formation of specific sanguinarine-BSA complex was further confirmed by monitoring the fluorescence changes of sanguinarine upon binding to the protein. The SI and SA forms have emission maxima at 564 and 420 nm, respectively, when excited at 475 and 327 nm [41]. On titrating with BSA, the fluorescence intensities of both forms quenched and attained saturation (see Figure 3 C & 3D). The binding has been quantified in terms of Stern-Volmer quenching plots. The *K*_sv_ values obtained from this experiment for iminium and alkanolamine binding to BSA were 7.2×10^4^ and 3.5×10^5^ L. mol^−1^, respectively. These values are in close agreement with the *K*_sv_ values obtained from the fluorescence quenching of the protein by sanguinarine (*vide supra*).

We examined the absorption spectra of the fluorophores in an additional experiment to confirm the static quenching mechanism. Since dynamic quenching affects only the excited states of the fluorophores, changes in the absorption spectra are not expected [38]. The difference absorption spectrum of sanguinarine and BSA in the complex state was recorded. The absorption spectra of BSA in presence of SI and SA forms are presented in Figure S1. Two absorption bands were observed at 278 nm and 328 nm. Sanguinarine iminium has absorption peaks in the same place as BSA (cf. Figure S1). The change in the absorbance at 278 nm is indicative of the influence of sanguinarine on the BSA spectrum rather than a simple spectral overlap.

### Energy Transfer from BSA to Sanguinarine and Calculation of Binding Distance

**Figure 4:**
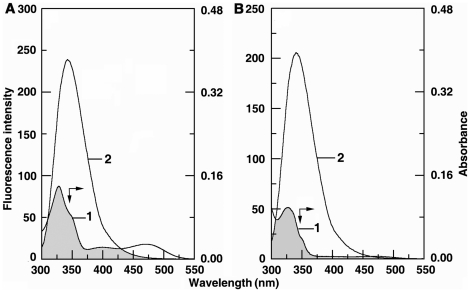
Spectral overlap (shaded portion) of sanguinarine absorption spectrum and BSA fluorescence spectrum at 25°C. In panel (A) and (B) curve 1 represent absorption spectra of iminium and alkanolamine form and curve 2 the fluorescence spectra of BSA. The ratio of the concentration of [BSA]∶[sanguinarine]) = 1∶1. The excitation of BSA was done at 295 nm.

The overlap (shaded) of the absorbance spectra of SI and SA forms with the fluorescence emission spectrum of BSA is shown in Figure 4A & 4B. The efficiency of energy transfer between sanguinarine and the Trp 212 residue of BSA can be used to evaluate the distance between them by Forster Resonance Energy transfer (FRET). Energy transfer could occur through direct electrodynamic interaction between the primarily excited molecules and its neighbors [42] that may happen when the donor can produce fluorescence light, when there is overlap between the fluorescence emission spectrum of the donor and the absorbance spectrum of the acceptor or when the distance between the donor and the acceptor is <8 nm [43]. By using the equation,
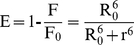
(3)where E is the efficiency of energy transfer, F and F_o_ are the fluorescence intensities in presence and absence of sanguinarine, and, R_o_ is the critical distance when the efficiency of transfer is 50%, R_o_ can be calculated using the equation,
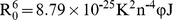
(4)where K^2^ is the spatial factor of orientation, n is the refractive index of the medium, and ϕ is the fluorescence quantum yield of the donor. J is the overlap integral of the fluorescence emission spectrum for the donor and the absorption spectrum of the acceptor (Figure 4), which may be calculated from the equation,
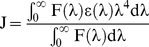
(5)where F(λ) is the fluorescence intensity of the donor at wavelength λ and ε(λ) is the molar absorption coefficient of the acceptor at wavelength λ. It was reported earlier [44] that for BSA n = 1.36 and p = 0.15. Accordingly from the above equation J = 2.131×10^−14^ cm^3^. L. mol^−1^, E = 0.27, R_o_ = 2.85 nm, and the binding distance r = 3.37 nm for sanguinarine iminium, and J = 1.286×10^−14^ cm^3^. L. mol^−1^, E = 0.64, R_o_ = 2.625 nm, and the binding distance r = 2.383 nm for the alkanolamine forms. So the distance between Trp residue at position 212 of BSA and bound sanguinarine, both SI and SA forms, is far lower than the 7 nm value and 0.5Ro<r<1.5Ro indicating high probability of energy transfer [43] from BSA to sanguinarine, in accordance with the static quenching mechanism. This result indicated the obeying of the conditions of Forster energy transfer theory for the binding, suggesting the location of both the forms of bound sanguinarine to be in domain II of BSA where the Trp 212 is located.

### Site selective binding of sanguinarine

**Figure 5:**
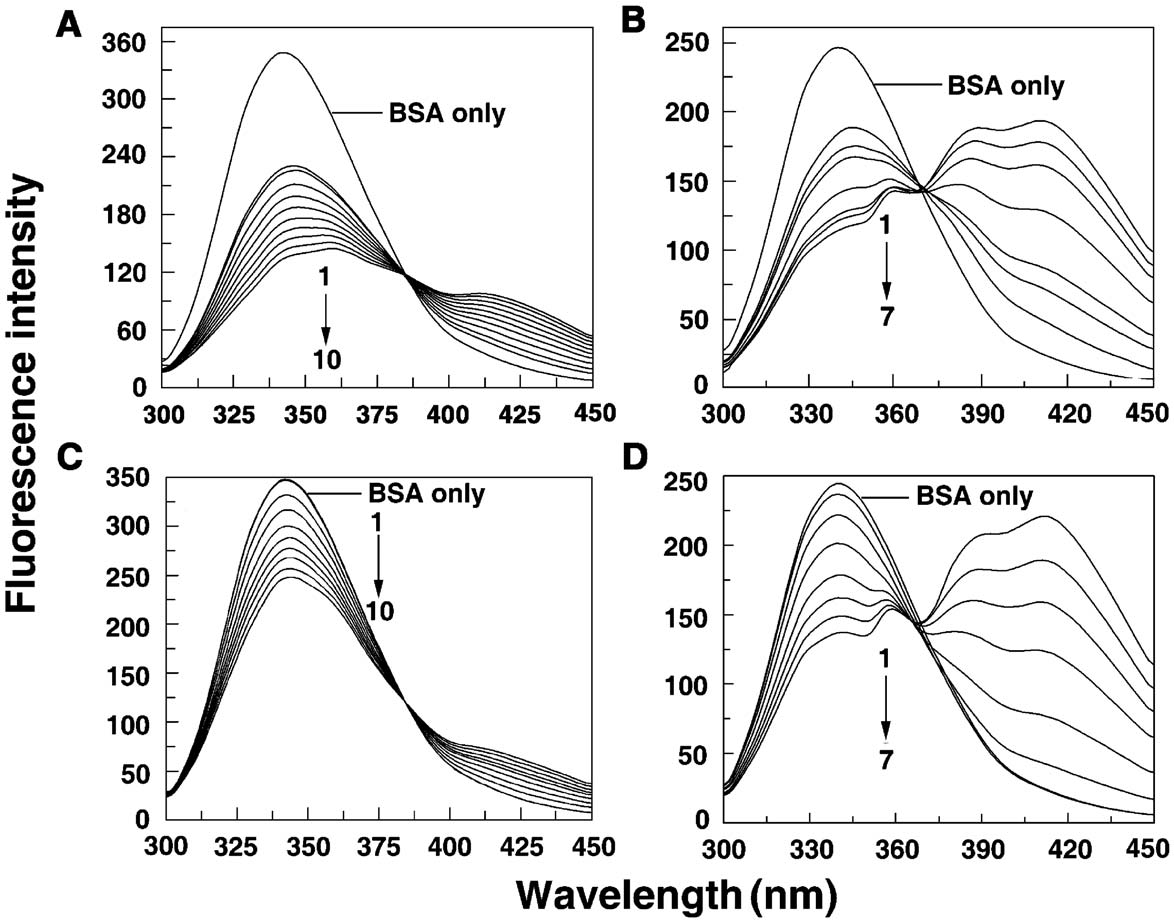
Effect of site marker on the sanguinarine-BSA complex (T = 298 K, λ_ex_ = 295 nm). C (warfarin) = C (BSA) = 5 µM with SI (panel A) and SA (panel B), respectively. C (ibuprofen) = C (BSA) = 5 µM with SI form (panel C) and SA (panel D), respectively. In panel (A) curves (1–10) correspond to 0, 0.5, 1.5, 2.5, 3.5, 4.5, 5.5, 6.5, 7.5 and 8.5 µM of SI and panel (C) curves (1–10) denote 0, 0.5, 1, 1.5, 2, 2.5, 3, 3.5, 4 µM of SA. Panel (B) and (D) curves (1–8) denote 0 0.5, 1.0, 1.5, 2, 2.5, 3, 3.5 µM of SA.

**Figure 6:**
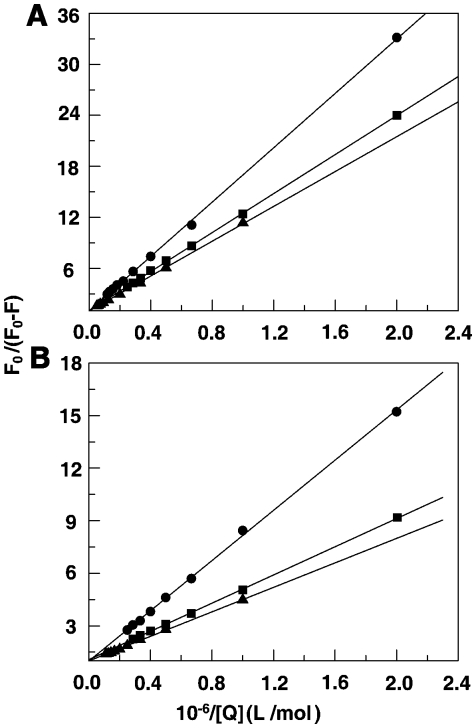
Modified Stern-Volmer plots of SI and SA of site marker competitive experiments. Respective modified Stern-Volmer plots of SI (panel A) and SA (panel B), blank (▴), in presence of warfarin (•) and in presence of ibuprofen (▪).

Site marker fluorescence probes warfarin and ibuprofen were used for monitoring site I and site II, respectively, of bovine serum albumin. Warfarin, an anticoagulant, explicitly binds to site I in the subdomain IIA while ibuprofen, a non steroidal anti-inflammatory agent, primarily binds to site II located in the subdomain IIIA [45]–[47]. Competitive binding experiments were carried out with BSA-warfarin and ibuprofen complexes in presence of SI and SA forms and the results are presented in Figure 5A & 5B. The emission maximum of BSA shifts from 341 to 344 nm on complexation with warfarin for both the sanguinarine forms. Addition of SI and SA forms quenched the fluorescence intensity of BSA. Figures 5 C & 5D present the comparative competitive binding pattern of ibuprofen-BSA complex with sanguinarine. In contrast to warfarin, the fluorescence intensity of ibuprofen-BSA complex was the same as that of free BSA and the binding of sanguinarine iminium and alkanolamine forms induced quenching as in the absence of ibuprofen. This indicated that sanguinarine binding was affected very little, if at all, in presence of ibuprofen. Thus, both SI and SA forms bind to site I in the subdomain IIA of BSA. In combination with the fluorescence quenching results it can be elicited that Trp 212 is near or within the binding site of sanguinarine. Several small molecules were found to bind to this location in BSA [48]–[53] and this site appears to be favourable for small molecules. To evaluate quantitatively the binding of sanguinarine in presence of these site markers, the binding constants in their presence were evaluated by modified Stern-Volmer equation
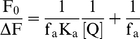
(6)where ΔF is the difference in fluorescence in the absence and presence of the quencher at concentration [Q], 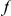
_a_ is the fraction of accessible fluorescence, and *K*_a_ is the effective quenching constant for the accessible fluorophores, which is similar to the binding constant for the quencher-acceptor systems. The dependence of *F*_0_/Δ*F* on [Q]^−1^) is linear and the slope is (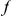
_a_K_a_)^−1^. Figure 6A & 6B shows the modified Stern-Volmer plots of BSA-sanguinarine complexation in presence of warfarin and ibuprofen. The results reveal that the binding, which was only marginally affected in presence of ibuprofen, was remarkably affected in presence of warfarin confirming the affinity of sanguinarine to Trp 212 of site I (subdomain IIA) of BSA. The values of the quenching constants for the complexes BSA-iminium and alkanolamine forms in presence and absence of the site markers are presented in Table 2.

**Table 2:** Variation in the binding constant of sanguinarine iminium and alkanolamine forms to BSA in presence of site markers ibuprofen and warfarin derived by the modified Stern-Volmer method at 25°C.

Sanguinarine	Site marker	*K*_a_ /10^−4^ L. mol^−1^
Iminium	Blank	9.75
	Ibuprofen	8.71
	Warfarin	6.25
Alkanolamine	Blank	28.0
	Ibuprofen	24.8
	Warfarin	13.9

The values are averages of four experiments. Experiments with iminium and alkanolamine forms were conducted in citrate-phosphate buffer, pH 6.4, and carbonate-bicarbonate buffer, pH 9.2, respectively.

### Circular dichroism spectroscopy

**Figure 7:**
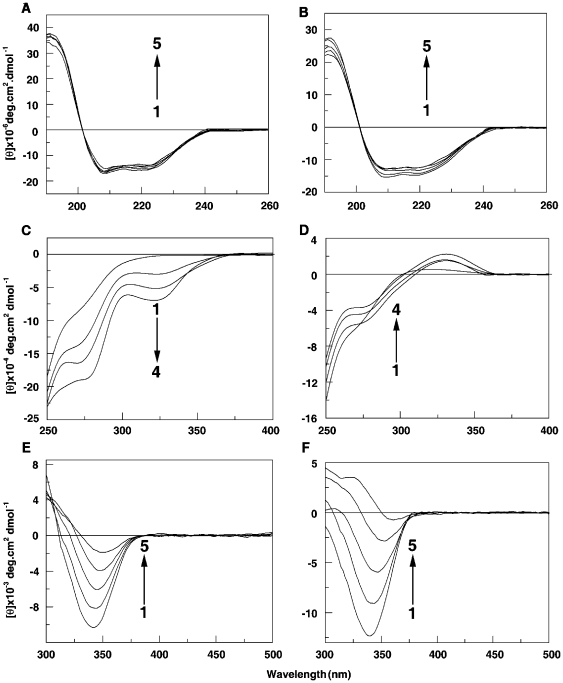
Intrinsic circular dichroic (CD far UV) spectral changes of BSA (1 µM) on interaction with SI (panel a) and SA (panel b). In panel (A) curves 1–5 denote 0, 10, 15, 25 and 35 µM of SI and panel (B) curves 1–5 denote 0, 4, 8, 12 and 22 µM of SA. Near UV CD spectral changes of BSA (2 µM) on interaction with SI (panel C) and SA (panel D). In panel (C) curves (1–4) denote 0, 10, 20, and 30 µM of SI and in panel (D) curves (1–4) denote 0, 20, 40, and 60 µM of SA. Induced CD spectra of SI (50 µM) (panel E) and SA (50 µM) (panel F) on interaction with BSA. Curves (1–5) in panel e denote 500, 400, 300, 200 and 100 µM and curves (1–5) in panel F denote 500, 400, 300, 200, and 100 µM of BSA, respectively.

Changes in the secondary and tertiary structure of the protein can be inferred from far and near ultraviolet CD studies The far uv CD spectrum of native BSA at both pH conditions showed two minima at 209 and 222 nm (curve 1 in Figure 7 A & B) which is typical of α-helical structure in accordance with the literature reports [54], [55]. The secondary protein conformation, determined by the Jasco software, was found to contain ∼56.0% α-helix, ∼30% β-sheet (both parallel and antiparallel) and ∼13% random coil conformation at both pH conditions (Table 3). Within the pH range 6.4–9.2 there is very little difference in the secondary and tertiary structures of the protein as evidenced from CD studies. Sanguinarine is not CD active. Upon titrating with increasing concentrations of sanguinarine iminium and alkanolamine forms, the CD spectrum of BSA decreased in intensity, indicating partial unfolding of the helical structure. There were significant differences in the extent of decrease in CD intensity by iminium and alkanolamine forms. At saturation corresponding to 35 and 22 µmols of iminium and alkanolamine forms, there was a reduction of α-helical structures to the extent of 16.4% and 18.3% (Table 3). Thus, the unfolding was slightly more with the alkanolamine form and at a much lower input ratio compared to the iminium form. Thus binding of both forms of sanguinarine induced secondary structural changes in BSA.

**Table 3:** Variation of the secondary structure of BSA in presence of different concentrations of sanguinarine iminium and alkanolamine forms at 25°C.

Sanguinarine	D/P	α-Helix(%)	β-Sheet(%)	Random coil(%)
Iminium	0	56.8	30.1	13.1
	10	55.9	30.7	13.4
	15	54.0	31.5	14.5
	25	47.5	34.7	17.8
	35	40.4	37.2	22.4
Alkanolamine	0	55.6	31.1	13.3
	4	53.4	34.0	12.6
	8	50.0	36.8	13.2
	12	46.1	37.4	16.5
	22	37.3	42.4	20.3

Based on the Figure 7A & B the data were analyzed by the Jasco software for secondary structure estimation. The experiments with iminium and alkanolamine forms were performed in citrate-phosphate buffer of pH 6.4 and carbonate-bicarbonate buffer of pH 9.2, respectively. D/P refers to drug/protein molar ratio.

The near UV CD spectrum of BSA showed two minima around 262 and 290 nm, characteristic of the disulphide and aromatic chromophores [56], [57], and reflected the tertiary structural organization of the protein. The ellipticity of the bands enhanced (Figure 7C) on binding of sanguinarine iminium form, while with the alkanolamine form they decreased slightly (Figure 7D). Although the differential behavior of iminium and alkanolamine forms on the near UV CD spectrum of the protein is not clear, the changes clearly indicate the occurrence of tertiary structural alteration leading to an unfolding of the native conformation. The tertiary structural perturbation of BSA was observed to be different with the cationic iminium compared to the neutral alkanolamine form.

On binding to the protein, both iminium and alkanolamine forms of sanguinarine, which are hitherto CD inactive acquired induced circular dichroism manifested by a negative band around 340 nm band (Figure 7E & 7F). The change in the induced CD on progression of the binding was similar but more pronounced with alkanolamine form compared to the iminium. Thus, the strong asymmetric environment of the protein induces optical activity in the bound sanguinarine molecules, indicative of the strong and similar bound environment of both forms of the alkaloid on the protein.

### Calorimetric characterization of sanguinarine-BSA interaction

**Figure 8:**
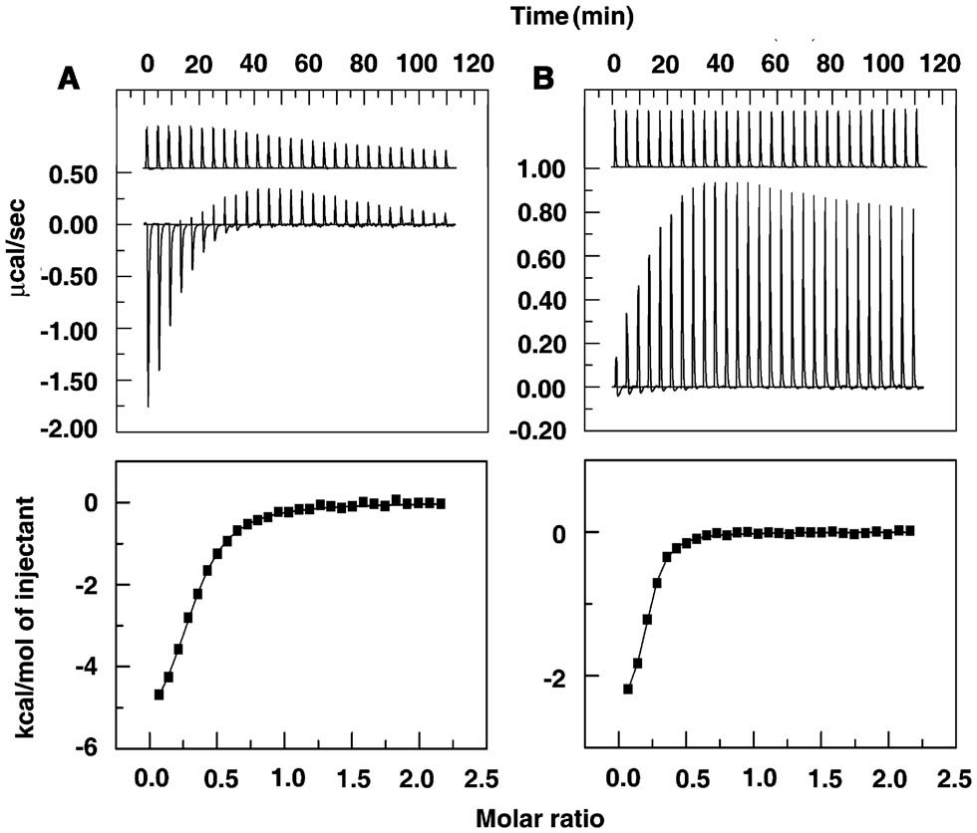
ITC profiles for the binding of sanguinarine to BSA. The top panels (A) and (B) present raw results for the sequential injection of SI (2.0 mM) and SA (1 mM) into BSA solution (0.2 and 0.1 mM) and dilution of sanguinarine into buffer (curves on the top offset for clarity). The lower panels show the integrated heat results after correction of heat of dilution against the mole ratio of sanguinarine/BSA. The points (closed squares) were fitted to a one-site model and the solid lines represent the best-fit results.

The interaction of the two forms of sanguinarine with BSA was investigated from ITC studies. ITC measurements provide detailed information [58] on thermodynamic quantities like enthalpy of binding (Δ*H*), the entropy contribution to the binding (*T*Δ*S*) and also the affinity and stoichiometry that can be correlated to the results of other experiments. Figure 8A & 8B (upper panels) shows representative calorimetric profiles of the titration of sanguinarine iminium and alkanolamine forms to BSA. The binding is exothermic in both the cases. The heat liberated in each injection was corrected for the heat of dilution that was determined in a separate but identical experiment injecting sanguinarine into buffer alone. The resulting values were plotted against the molar ratio of sanguinarine/BSA and fitted to a one-site model by nonlinear least square method (curves in the lower panel). The equilibrium constant (*K*_b_), binding stoichiometry (N), enthalpy change (Δ*H*), entropy contribution (*T*Δ*S*) and the free energy change obtained from the calorimetric data are summarized in Table 4. The binding constants of sanguinarine iminium and alkanolamine forms to BSA are revealed to be 7.94×10^4^ M^−1^ and 3.78×10^5^ M^−1^, respectively. These values are fairly close to the binding affinity values obtained from fluorescence titration data (*vide infra*). The number of binding sites for alkanolamine (0.197) was smaller compared to that for the iminium (0.298). The Gibbs energy change for the binding of the alkanolamine form was higher than that of the iminium by about 1.3 kcal/mol. The energetics of the interaction was also significantly different in the two cases. The binding of the iminium was clearly enthalpy driven (Δ*H* = −6.148 kcal/mol) with a small entropy contribution (*T*Δ*S* = 0.320 kcal/mol), while that of the alkanolamine form was driven by enthalpy changes (−2.557 kcal/mol) and a strong entropic component (*T*Δ*S* = 5.125 kcal/mol) that was favorable to the binding. The large value of enthalpy change for SI compared to SA is indicative of a stronger electrostatic interaction of the former with BSA and is due to the charge on the nitrogen atom.

**Table 4:** Temperature dependent isothermal titration calorimetric data for the binding of iminium and alkanolamine forms of sanguinarine to BSA.

Sanguinarine	*T*(K)	N	*K_b_ /*10^4^ M^−1^	Δ*G/*kcal mol^−1^	Δ*H/*kcal mol^−1^	*T*Δ*S/*kcal mol^−1^	Δ*C_p_ /*cal mol K
Iminium	288	0.349	9.44	−6.665	−3.295	3.370	−287
	298	0.298	7.94	−6.468	−6.148	0.320	
	308	0.272	5.08	−6.638	−9.048	−2.410	
Alkanolamine	288	0.182	70.7	−7.701	−1.193	6.508	−148
	298	0.197	37.8	−7.682	−2.557	5.125	
	308	0.185	14.3	−7.553	−4.165	3.388	

All the data in this table are derived from ITC experiments and are average of four determinations. *K_b_* and Δ*H* values were determined from ITC profiles fitting to Origin 7 software. The values of Δ*G* and *T*Δ*S* were determined using the equations Δ*G = −RTlnK_b_* and *T*Δ*S* = Δ*H*−Δ*G*. All the ITC profiles were fit to a model of single binding site. The experiments with iminium and alkanolamine forms were performed in citrate-phosphate buffer of pH 6.4 and carbonate-bicarbonate buffer of pH 9.2, respectively.

### Temperature dependent calorimetry results

To obtain insight into the forces driving the interaction between sanguinarine and BSA, complex formation was examined as a function of temperature (15–35°C). The binding isotherms observed at different temperatures were sigmoidal (not shown). Overall, as the temperature was increased, the binding enthalpies were negative with their magnitudes increasing. The negative enthalpy of binding at all temperatures indicated favorable exothermic binding interaction between sanguinarine and the protein. In case of SI, the entropy contribution became more and more unfavorable with increasing temperature. Although the entropy contribution decreased with increasing temperature in the case of SA, it remained favorable for the binding even at 35°C. This suggests that the binding of the iminium was driven by both enthalpy and entropy at lower temperature, but the enthalpy of binding becomes the dominant force at higher temperatures. With SA on the other hand, the binding that was entropy dominated at lower temperatures became enthalpy dominated at higher temperature. The energetics of the interaction indicated significant differences in the molecular forces that contribute and control the binding of iminium and alkanolamine forms to BSA.

**Figure 9:**
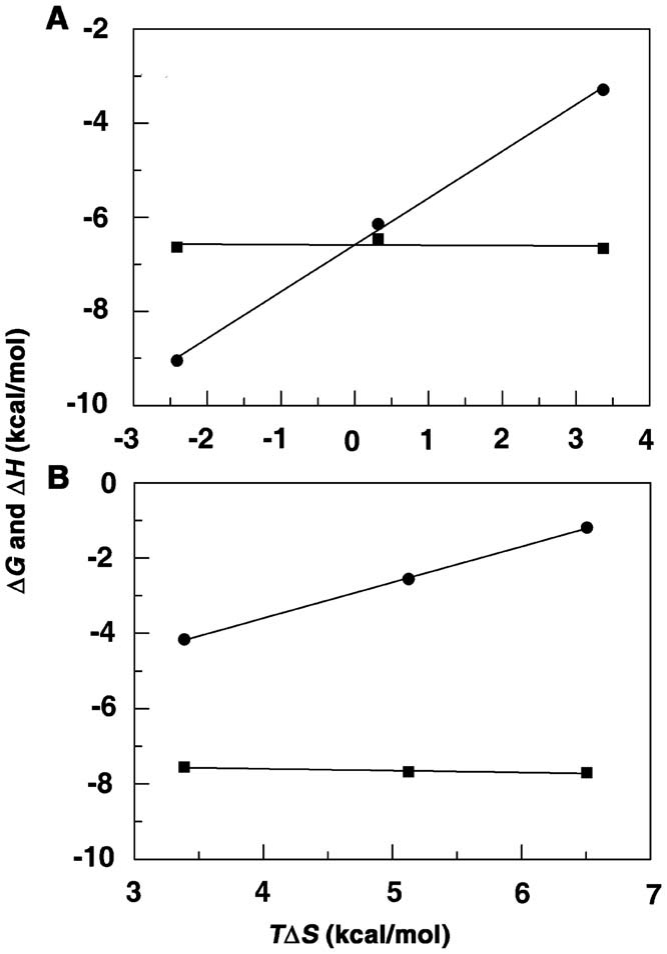
Plots of variation of thermodynamic parameters. Δ*G* (▪) and Δ*H* (•) versus *T*Δ*S* for the binding of sanguinarine iminium (A) and alkanolamine (B) forms with BSA.

**Figure 10:**
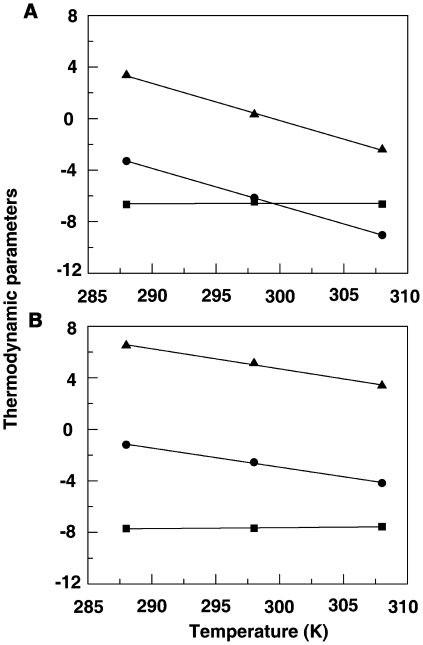
Plot of the thermodynamic binding parameters of sanguinarine-BSA complexation against temperature. *T*Δ*S* (▴-▴), Δ*H* (•-•) and Δ*G* (▪-▪) for iminium form (panel A) and alkanolamine form (panel B).

From the variation of the enthalpy with temperature, ITC data provides information on the heat capacity changes of the binding [59]. The observed enthalpy varied linearly with the experimental temperature in the range 15–35°C for both iminium and alkanolamine forms indicating that there is no measurable shift in the preexisting equilibrium between the conformational states of BSA in the temperature range studied. Strong enthalpy-entropy compensation was however observed, making Gibbs energy (Δ*G*) of binding nearly independent of temperature (Figure 9A & 9B). To determine the change in heat capacity (Δ*C_p_*), namely the first derivative of temperature dependence of enthalpy change, the data were plotted as Δ*H* versus temperature (Figure 10 A & 10B) to yield Δ*C_p_* values of −287 and −148 cal mol K^−1^, respectively, for the binding of iminium and alkanolamine forms. The negative value of Δ*C_p_* in both cases indicates that the binding is specific and accompanied by burial of non polar surface area [60], [61]. In addition, the negative heat capacity also causes the shift from a predominantly entropy contributed binding to an enthalpy dominated one as the temperature rises. In case of iminium, the entropic contribution to the binding process is zero at 26.47°C while for alkanolamine it is zero at 57°C. These are the temperatures at which the binding process becomes completely driven by enthalpy. Again, from the linear fit of the binding enthalpy versus temperature, the net enthalpic contribution for the binding of SI and SA is ∼0 kcal/mol at 3.57 and 7.24°C, respectively, with the binding process completely driven by entropy. Thus, between 3.57 and 26.47°C for SI and 7.24 and 57°C for SA, the complex formation was favored by both enthalpy and entropy contributions to the Gibbs energy. The temperature dependence of the binding constants for the formation of BSA-SI complex showed linear behavior while the same for BSA-SA complex showed non-linear behavior (not shown). The non-linear behavior for the latter may indicate contributions from linkage interactions or conformational changes during the binding process. It is pertinent to note that a stronger conformational change was observed for the binding of alkanolamine form by CD studies (*vide supra*). Furthermore, electrostatic interactions are known to contribute a positive term to the heat capacity changes and this may be the reason for the lower value of Δ*C_p_* for the charged species SI compared to the neutral species SA. A higher negative Δ*C_p_* for SA may also indicate a larger contribution from other binding coupled events than that from surface burial. This may involve strong H-bonding by the OH at the C6 position of SA to amino acids of BSA. Such an H-bonding has been shown to occur in sanguinarine-reductase-SA interactions [62]. A detailed analysis of the various contributions to Δ*C_p_* is beyond the scope of this paper and is not attempted.

### Conclusions

A study on the interaction of bovine serum albumin with cationic and neutral forms of the benzophenanthridine alkaloid sanguinarine has been performed. Absorbance and fluorescence quenching studies provide evidence for the formation of strong complexes. The binding parameters calculated from Stern-Volmer quenching method revealed that the affinity to BSA was higher for the neutral alkanolamine form compared to the charged iminium form. Specific distances of 3.37 and 2.38 nm, respectively, between Trp 212 and sanguinarine iminium and alkanolamine forms were obtained from FRET studies. Thus, the iminium form is bound closer to the Trp compared to alkanolamine form. Competitive binding using site markers demonstrated that the binding of both iminium and alkanolamine forms was at site I (subdomain II) of BSA. Sanguinarine binding altered the protein conformation by partially unfolding the structure; this was again more pronounced with the alkanolamine form. Hydrophobic interaction and van der Waals contacts along with H-bonding by the −OH group may contribute to the stronger binding of SA to BSA. The alkanolamine form is more hydrophobic compared to iminium. Furthermore, the π electrons of the aromatic SA ring system may contribute negative charges that can also contribute as electrostatic interaction with the positive charged sites available in the protein. Thermodynamic studies suggest that the binding was enthalpy driven for SI but was favoured by both negative enthalpy and strong favourable entropy contributions with SA form. The predominant hydrophobicity effect is clearly evident from the relatively large entropy contribution for the alkanolamine form. Electrostatic interaction may be the dominant force in the iminium binding. This notion got strengthened from the fact that there is a large enthalpic component for the binding of iminium suggestive of forces other than hydrophobic effect to contribute significantly to the binding. This must essentially come from electrostatic, van der Waals and H-bonding interactions. The results of binding to serum protein presented here are interesting in the light of the strong binding of the iminium form and the complete absence of binding of the alkanolamine form with nucleic acids.

## Materials and Methods

### Biochemicals

Bovine serum albumin (BSA), sanguinarine, warfarin, ibuprofen and all buffer salts were products of Sigma-Aldrich (St. Louis, MO, USA).

### Preparation of stock solutions

The samples were prepared in 10 mM citrate-phosphate buffer of pH 6.4 and 10 mM carbonate-bicarbonate buffer of pH 9.2. Concentrations were determined by UV absorbance measurements using molar extinction coefficients of 43,824 M^−1^ cm^−1^ at 280 nm for BSA, 3.07×10^4^ M^−1^ cm^−1^ at 327 nm for iminium and 2.16×10^4^ M^−1^ cm^−1^ for alkanolamine form, respectively. Experiments with iminium and alkanolamine forms were conducted in citrate-phosphate buffer, pH 6.4, and carbonate-bicarbonate buffer, pH 9.2, respectively.

### Absorbance spectroscopy

Absorbance spectral measurements were performed on a Jasco V660 double beam double monochromator spectrophotometer (Jasco International Co., Ltd. Tokyo, Japan) in 1 cm path length strain-free matched quartz cells. Temperature of the cell holder was controlled by a Lab companion water bath.

### Fluorescence spectroscopy

Steady state fluorescence spectra were measured on a Shimadzu RF5301PC spectrofluorimeter (Shimadzu Corporation, Kyoto, Japan) using excitation and emission band pass of 3 nm. For measurement of intrinsic fluorescence of BSA in presence of sanguinarine, the protein sample was excited at 295 nm, the excitation maximum of tryptophan, and emission spectra scanned from 300 to 400 nm. In presence of BSA, iminium and alkanolamine forms were excited at 475 and 327 nm, respectively. Temperature dependent fluorescence spectral studies were conducted on a Hitachi F4010 unit (Hitachi Ltd., Tokyo, Japan) equipped with a Eyela Uni cool water bath (Tokyo Rikakikai Co., Tokyo, Japan) for controlling the sample temperature. The temperature was monitored by the electronic devise Sensortek, model BAT-12 (Sensortek Inc., NJ, USA).

### Circular dichroism (CD) spectroscopy

A Jasco J815 spectropolarimeter (Jasco International Co., Ltd. Tokyo, Japan) equipped with a peltier cell holder and temperature controller PFD425 L/15 was used for monitoring the conformational changes in the protein on alkaloid binding. The protein concentration and path length of the cell used were 1.0 µM and 0.1 cm for far UV CD and 1.0 µM and 1 cm for near UV CD, respectively. The instrument parameters for CD measurements were scanning speed of 50 nm/min., bandwidth of 1.0 nm, and sensitivity of 100 milli degree. Four scans were averaged and smoothed to improve signal-to-noise ratio. The molar ellipticity values are expressed in terms of mean residue molar ellipticity [θ], in units of deg. cm^2^ dmol^−1^ taking a value of 113 as mean residue weight for BSA. Secondary structure analysis was performed by the software supplied by Jasco.

### Isothermal titration calorimetry

The energetics of the binding of sanguinarine to BSA was studied by isothermal titration calorimetry (ITC) using a VP ITC unit (MicroCal, Northamption, MA, USA). The calorimeter cell had a volume of 1.423 mL. All solutions were degassed under vacuum (140 mbar, 10 min) on the Microcal's Thermovac unit to eliminate air bubbles before loading. The instrument control, titration and analysis were performed through dedicated Origin 7 software provided with the unit. The titration experiments were carried out as follows. The calorimeter syringe was filled with a concentrated solution of the alkaloid (2 mM for the iminium form and 1 mM for the alkanolamine form). Successive injections of 10 µL of this solution into 0.2 mM and 0.1 mM solutions of BSA, respectively, in the same buffer contained in the calorimeter cell was effected from the rotating syringe that enabled constant stirring of the solution. The data were corrected for the heat of dilution of sanguinarine, which was determined in a separate set of experiments under identical conditions.

## Supporting Information

Figure S1 Difference spectrum of BSA and sanguinarine iminium (A) and alkanolamine forms (B).
(TIF)
